# An Adaptive Moving Target Imaging Method for Bistatic Forward-Looking SAR Using Keystone Transform and Optimization NLCS

**DOI:** 10.3390/s17010216

**Published:** 2017-01-23

**Authors:** Zhongyu Li, Junjie Wu, Yulin Huang, Haiguang Yang, Jianyu Yang

**Affiliations:** 1 School of Electronic Engineering, University of Electronic Science and Technology of China, No. 2006, Xiyuan Ave, West Hi-Tech Zone, Chengdu 611731, China; junjie_wu@uestc.edu.cn (J.W.); yulinhuang@uestc.edu.cn (Y.H.); yanghaiguang@uestc.edu.cn (H.Y.); jyyang@uestc.edu.cn (J.Y.); 2 Department of Information Engineering, Electronics and Telecommunications (DIET), University of Rome “La Sapienza”, Via Eudossianan, n.18, cap 00184 Roma, Italy

**Keywords:** bistatic forward-looking SAR, moving-target imaging, adaptive, keystone transform, optimization

## Abstract

Bistatic forward-looking SAR (BFSAR) is a kind of bistatic synthetic aperture radar (SAR) system that can image forward-looking terrain in the flight direction of an aircraft. Until now, BFSAR imaging theories and methods for a stationary scene have been researched thoroughly. However, for moving-target imaging with BFSAR, the non-cooperative movement of the moving target induces some new issues: (I) large and unknown range cell migration (RCM) (including range walk and high-order RCM); (II) the spatial-variances of the Doppler parameters (including the Doppler centroid and high-order Doppler) are not only unknown, but also nonlinear for different point-scatterers. In this paper, we put forward an adaptive moving-target imaging method for BFSAR. First, the large and unknown range walk is corrected by applying keystone transform over the whole received echo, and then, the relationships among the unknown high-order RCM, the nonlinear spatial-variances of the Doppler parameters, and the speed of the mover, are established. After that, using an optimization nonlinear chirp scaling (NLCS) technique, not only can the unknown high-order RCM be accurately corrected, but also the nonlinear spatial-variances of the Doppler parameters can be balanced. At last, a high-order polynomial filter is applied to compress the whole azimuth data of the moving target. Numerical simulations verify the effectiveness of the proposed method.

## 1. Introduction

Forward-looking imaging has many potential applications. For example, it can be used in airplane navigation and landing, independent of weather conditions and the time of the day. With the usual monostatic synthetic aperture radar (SAR) configuration, forward looking mode cannot form a 2-D image, because the directions of the Doppler and range resolutions are the same at the area of the forward-looking direction [1,2].

Currently, as an emerging SAR technology, bistatic SAR (BiSAR) has received considerable attention [3,4,5,6]. In BiSAR, the transmitter and receiver are placed on different platforms; therefore, the two resolution directions are determined both by the transmitter and receiver. When one platform works in the forward-looking mode, the other can insure the difference between the two resolution directions, thus generating a 2-D image [1,7]. This kind of BiSAR is called bistatic forward-looking SAR (BFSAR) [8,9,10]. The advantages of BFSAR have been analyzed in [1,11,12], and the forward-looking imaging ability of airborne BFSAR has been first tested by us in 2013 [10].

The primary processing steps for BFSAR imaging include a range cell migration (RCM) correction step and an azimuth compression step [13,14,15]. For static target imaging, the RCM as well as azimuth Doppler parameters are only contributed to by the ascertained BFSAR platform movements. That is to say, the RCM correction (RCMC) and azimuth compression can be accurately accomplished based on the geometry information between the BFSAR platforms and the static target. However, for moving-target imaging, coupling effect between the non-cooperative moving-target movement and the platform movements induces unknown RCM and spatial-variant Doppler parameters. It should be pointed out that the main drawbacks in exploiting BFSAR for moving-target imaging include (I) large and unknown RCM (including range walk and high-order RCM); (II) the spatial-variances of the Doppler parameters (including Doppler centroid and high-order Doppler) are not only unknown, but also nonlinear for different point-scatterers of the target. The moving target is smeared and shifted in the BFSAR image if conventional imaging algorithms are applied.

Nevertheless, the extant literature and reports on BFSAR imaging theories are mainly focused on stationary scenario imaging, such as the range doppler (RD) algorithm [13], nonlinear chirp scaling (CS) algorithms [14,15] and Omega-k algorithms [16,17]. In [18,19], BFSAR moving-target detection and imaging was studied first, and a detection method based on product second-order ambiguity function in the Doppler frequency rate (DFR) domain is proposed. However, only the DFR can be estimated here, which causes azimuth displacement since the Doppler centroid cannot be estimated. In order to simultaneously estimate the DFR and Doppler centroid, an imaging method based on mismatched compression was proposed in [20,21]. The main shortcomings of the existing BFSAR moving-target imaging methods in [18,19,20,21] include three aspects: (I) only the range walk can be corrected, while the high-order RCM cannot be compensated, which cannot be ignored for high-resolution and high-precision imaging; (II) the nonlinear spatial-variances of the Doppler parameters cannot be compensated, thus causing azimuth dislocation and shape distortion; (III) The third-order Doppler parameter also cannot be estimated by the existing methods. Thus, moving-target imaging continues to be an issue that needs to be resolved for BFSAR.

In this paper, an adaptive moving-target imaging method, which is based on keystone transform and optimization NLCS, is proposed for BFSAR. This method relies on a proper processing of the data aiming at, first, to correct the range walk by applying keystone transform over the whole received echo, and then, the relationships among the unknown high-order RCM, the nonlinear spatial-variances of the Doppler parameters, and the speed of the mover, are established. After that, the moving-target imaging problem is transformed to be a new optimization problem. In order to get a higher efficiency, differential evolution (DE), which is arguably one of the most powerful stochastic real-parameter optimization algorithms in current use, is applied to solve the new optimization problem. Particularly, the NLCS processing includes third/fourth-order filtering processing in the azimuth frequency domain, and second/third/fourth-order azimuth chirp scaling processing in the azimuth time domain. Associated with the aforesaid five freedoms, not only the spatial-variant Doppler centroid, but also the nonlinear spatial-variant high-order Doppler can be balanced simultaneously. Finally, a high-order polynomial filter is applied to compress the whole azimuth data. Compared with the existing BFSAR moving-target imaging methods, the improvements of this method include two main aspects: (I) not only the range walk but also the unknown high-order RCM can be corrected simultaneously; (II) the nonlinear spatial-variances of the Doppler parameters associated to different point-scatterers can be compensated, thus avoiding azimuth dislocation and shape distortion.

The remainder of this paper is organized as follows. In Section 2, we first describe the operative conditions of the BFSAR system and derive the signal model of the moving target, followed by an analysis of the basic echo properties of the moving target. In Section 3, the adaptive moving-target imaging method based on keystone transform and optimization NLCS is described in detail. Numerical simulations are given in Section 4. Finally, Section 5 concludes this paper.

## 2. Signal Model

**Figure 1:**
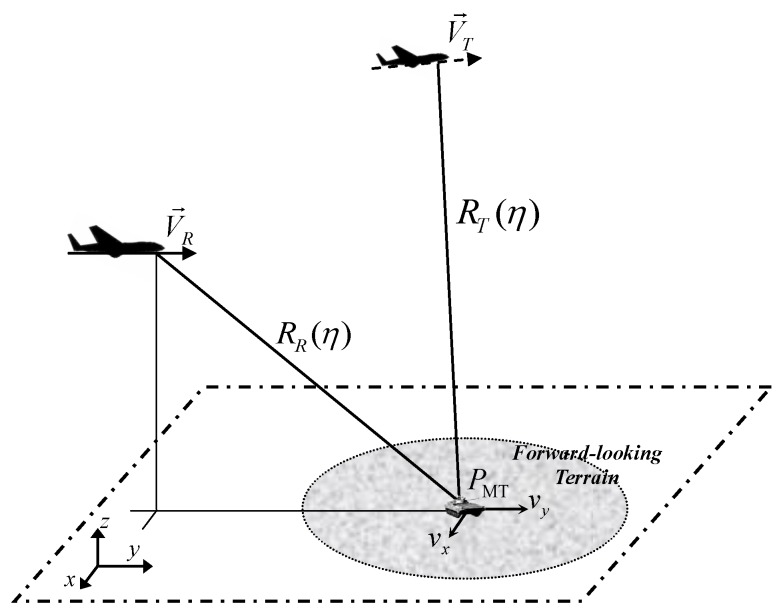
Geometrical relationship between aircrafts and the moving target for BFSAR.

Here, BFSAR imaging geometrical configuration for a moving target is constructed first and then the signal model is shown, giving the Doppler parameters (including Doppler centroid, DFR and third-order Doppler parameter). The geometrical relationship between the aircraft and the moving target is shown in Figure 1. In the three dimensional (3-D) coordinate system, the *x*-*y* plane defines the surface of the Earth and the *z*-axis points away from the Earth. ${\vec{V}}_{R}$ and ${\vec{V}}_{T}$ denote the velocity vectors of the receiver and transmitter, respectively. Supposing that the flight direction of the receiver is parallel to the *y*-axis, while the transmitter has an angle *α* with the *y*-axis. Supposing that the original coordinate of the receiver is $\left(x_{R},y_{R},z_{R}\right)$, and the transmitter is $\left(x_{T},y_{T},z_{T}\right)$. $P_{MT}$ is a moving target, whose original coordinate is (x,y,0). Supposing that $v_{x}$ and $v_{y}$ denote the cross- and along-track velocities of the moving target, respectively. $R_{R}\left(\eta \right)$ and $R_{T}\left(\eta \right)$ represent the instant slant ranges from the receiver platform and the transmitter platform to $P_{MT}$, respectively. *η* is the azimuth slow time variable.

The slant range history with respect to the point-scatterer $P_{MT}\left(x,y\right)$ of the receiver is given by

(1) $$R_{R}\left(\eta ;x,y\right)=\sqrt{{\left[x_{R}-x-v_{x}\eta \right]}^{2}+{\left[y_{R}+V_{R}\eta -y-v_{y}\eta \right]}^{2}+z_{R}^{2}}\approx R_{cR}+A_{r}\eta +\frac{1}{2}B_{r}\eta^{2}+\frac{1}{6}C_{r}\eta^{3}$$

(2) $$\begin{array}{cc} & A_{r}\left(x,y\right)=\frac{-\left(x_{R}-x\right)v_{x}\;+\;\left(y_{R}-y\right)\left(V_{R}-v_{y}\right)}{R_{cR}} \end{array}$$

(3) $$\begin{array}{cc} & B_{r}\left(x,y\right)=\frac{v_{x}^{2}+{\left(V_{R}-v_{y}\right)}^{2}}{R_{cR}}-\frac{\left[-v_{x}\left(x_{R}-x\right)\;+\;\left(y_{R}-y\right)\left(V_{R}-v_{y}\right)\right]A_{r}}{R_{cR}^{2}} \end{array}$$

(4) $$\begin{array}{cc} C_{r}\left(x,y\right) & =-\frac{2\left[v_{x}^{2}+{\left(V_{R}-v_{y}\right)}^{2}\right]A_{r}}{R_{cR}^{2}}-\frac{\left[-v_{x}\left(x_{R}-x\right)\;+\;\left(y_{R}-y\right)\left(V_{R}-v_{y}\right)\right]B_{r}}{R_{cR}^{2}}\\ & \;+\frac{2{\left[-v_{x}\left(x_{R}-x\right)\;+\;\left(y_{R}-y\right)\left(V_{R}-v_{y}\right)\right]}^{2}A_{r}}{R_{cR}^{4}} \end{array}$$

where $R_{cR}$ represents the squint distance of the receiver from $P_{MT}\left(x,y\right)$ at beam center time. $V_{R}=\left|{\vec{V}}_{R}\right|$.

In addition, the slant range history of the transmitter is given by

(5) $$R_{T}\left(\eta ;x,y\right)=\sqrt{{\left[x_{T}\;+\;V_{Tx}\eta -x-v_{x}\eta \right]}^{2}+{\left[y_{T}+V_{Ty}\eta -y-v_{y}\eta \right]}^{2}+z_{T}^{2}}\approx R_{cT}+A_{t}\eta +\frac{1}{2}B_{t}\eta^{2}+\frac{1}{6}C_{t}\eta^{3}$$

(6) $$\begin{array}{cc} & A_{t}\left(x,y\right)=\frac{\left(x_{T}-x\right)\left(V_{Tx}-v_{x}\right)\;+\;\left(y_{T}-y\right)\left(V_{Ty}-v_{y}\right)}{R_{cT}} \end{array}$$

(7) $$\begin{array}{cc} & B_{t}\left(x,y\right)=\frac{{\left(V_{Tx}-v_{x}\right)}^{2}+{\left(V_{Ty}-v_{y}\right)}^{2}}{R_{cT}}-\frac{\left[\left(V_{Tx}-v_{x}\right)\left(x_{T}-x\right)\;+\;\left(y_{T}-y\right)\left(V_{Ty}-v_{y}\right)\right]A_{t}}{R_{cT}^{2}} \end{array}$$

(8) $$\begin{array}{cc} C_{t}\left(x,y\right) & =-\frac{2\left[{\left(V_{Tx}-v_{x}\right)}^{2}+{\left(V_{Ty}-v_{y}\right)}^{2}\right]A_{t}}{R_{cT}^{2}}-\frac{\left[\left(V_{Tx}-v_{x}\right)\left(x_{T}-x\right)\;+\;\left(y_{T}-y\right)\left(V_{Ty}-v_{y}\right)\right]B_{t}}{R_{cT}^{2}}\\ & \;+\frac{2{\left[\left(V_{Tx}-v_{x}\right)\left(x_{T}-x\right)\;+\;\left(y_{T}-y\right)\left(V_{Ty}-v_{y}\right)\right]}^{2}A_{t}}{R_{cT}^{4}} \end{array}$$

where $R_{cT}$ represents the squint distance of the transmitter from $P_{MT}\left(x,y\right)$ at beam center time, $V_{Tx}=-V_{T}\sin\alpha$, $V_{Ty}=V_{T}\cos\alpha$, and $V_{T}=\left|{\vec{V}}_{T}\right|$.

After demodulation of the baseband, the received moving target echo can be written as follows in terms of azimuth time *η* and range time *τ*, which is given by

(9) $$\begin{array}{c} S\left(\eta ,\tau ;x,y\right)=\sigma S_{t}\left(\tau -\frac{R_{R}\left(\eta ;x,y\right)+R_{T}\left(\eta ;x,y\right)}{c}\right)\times \omega \left(\eta \right)\exp\left[-j2\pi \frac{R_{R}\left(\eta ;x,y\right)+R_{T}\left(\eta ;x,y\right)}{\lambda }\right] \end{array}$$

where *σ* denotes the scattering coefficient of the target, ω(η) the azimuth antenna pattern, $S_{t}\left(\tau \right)$ the transmitted baseband signal (supposing it is a LFM signal here), *c* the speed of the electromagnetic wave, and *λ* the carrier wavelength. The last exponential term is the azimuth Doppler term, which includes the modulated information of the azimuth signal.

The Doppler frequency is the first-order derivative of the Doppler phase term in Equation (9) versus the azimuth time *η*, which can be expressed as

(10) $$\begin{array}{c} \begin{array}{c} f_{d}\left(\eta ;x,y\right)=-\frac{1}{\lambda }\frac{d\left[R_{R}\left(\eta ;x,y\right)+R_{T}\left(\eta ;x,y\right)\right]}{d\eta }\approx f_{dc}\left(x,y\right)+f_{dr}\left(x,y\right)\eta +\frac{1}{2}f_{d3}\left(x,y\right)\eta^{2} \end{array} \end{array}$$

where $f_{dc}\left(x,y\right)$ is the Doppler centroid, $f_{dr}\left(x,y\right)$ denotes the DFR, and $f_{d3}\left(x,y\right)$ represents the third-order Doppler parameter. The analytical expressions of the above Doppler parameters can be obtained from Equation (1) to (8), which are given by

(11) $$\begin{array}{c} f_{dc}\left(x,y\right)=-\frac{A_{r}\left(x,y\right)+A_{t}\left(x,y\right)}{\lambda } \end{array}$$

(12) $$\begin{array}{c} f_{dr}\left(x,y\right)=-\frac{B_{r}\left(x,y\right)+B_{t}\left(x,y\right)}{\lambda } \end{array}$$

(13) $$\begin{array}{c} f_{d3}\left(x,y\right)=-\frac{C_{r}\left(x,y\right)+C_{t}\left(x,y\right)}{\lambda } \end{array}$$

Generally, moving-target velocity parameters are unknown, whereas the foregoing analysis reveals that both RCM and azimuth modulation are all related to them. Therefore, BFSAR moving-target imaging requires correction of the unknown RCM in Equations (1) and (5) (including range walk and range curvature), as well as compensation for the additional modulation of the azimuth polynomial-phase signal (including Doppler centroid, DFR and third-order Doppler parameter, where the wrong Doppler centroid causes displacement and the wrong DFR and third-order Doppler parameter cause defocusing).

More difficult, according to Equations (11)–(13), we can observe that the Doppler parameters are related to the coordinate of the point-scatterer. That means that different point-scatterers of the moving target have different Doppler parameters, and such a problem is called spatial-variances of the Doppler parameters. For the moving target with BFSAR, the spatial-variances of the Doppler parameters are not only unknown, but also nonlinear for different point-scatterers.

In the succeeding section, an adaptive moving-target imaging method, which is based on keystone transform and optimization NLCS, for BFSAR is analyzed in detail.

## 3. Moving Target Imaging Method

Associating Equation (1) with (5), the BFSAR bistatic range history for $P_{MT}\left(x,y\right)$ can be given by,

(14) $$\begin{array}{c} R_{R}\left(\eta ;x,y\right)+R_{T}\left(\eta ;x,y\right)\approx R_{bs}\left(0;x,y\right)+A\eta +\frac{B}{2}\eta^{2}+\frac{C}{6}\eta^{3} \end{array}$$

where $R_{bs}\left(0;x,y\right)=R_{cR}+R_{cT}$, $A=A_{r}+A_{t}$, $B=B_{r}+B_{t}$, $C=C_{r}+C_{t}$. $A_{r}$, $B_{r}$ and $C_{r}$ are given in Equations (2)–(4), respectively. $A_{t}$, $B_{t}$ and $C_{t}$ are given in Equations (6)–(8), respectively.

After the slant range expansion in Equation (14), the phase term in Equation (9) after range FFT is given by

(15) $$\begin{array}{c} \begin{array}{c} \varphi \left(\eta ,f_{\tau }\right)=-\pi \frac{f_{\tau }^{2}}{K_{r}}-\frac{2\pi }{c}\left(f_{\tau }+f_{c}\right)\left[R_{bs}\left(0\right)+A\eta +\frac{B}{2}\eta^{2}\;+\;\frac{C}{6}\eta^{3}\right] \end{array} \end{array}$$

where $K_{r}$ is the frequency rate of the transmitted LFM signal, $f_{c}$ is the carrier frequency. Notice that the second phase term is a linear phase of the range frequency variable $f_{\tau }$, so it represents a position shift of the echo signal in the range direction with *η* versus *τ* plane. If we construct a compensation factor in the range frequency and azimuth time domain using the geometric parameters of the stationary target, the RCM of the moving target cannot be fully compensated for because it ignores the movement of the moving target.

### 3.1. Range Walk Correction

To eliminate the Doppler ambiguity and transform the skewed spectrum into a quasi-orthogonal one, firstly the pre-filter is constructed as [20]

(16) $$\begin{array}{c} h_{\mathrm{filter}}=\exp\left[j\frac{2\pi }{c}\left(f_{\tau }+f_{c}\right)A_{ref}\eta \right] \end{array}$$

where $A_{ref}$ is the first-order coefficient of the bistatic range history for one reference stationary target.

After the pre-filtering, the filtered signal phase is given by

(17) $$\begin{array}{c} \begin{array}{c} \varphi_{1}\left(\eta ,f_{\tau }\right)=-\pi \frac{f_{\tau }^{2}}{K_{r}}-\frac{2\pi }{c}\left(f_{\tau }+f_{c}\right)\left[R_{bs}\left(0\right)+A^{\prime }\eta +\frac{B}{2}\eta^{2}\;+\;\frac{C}{6}\eta^{3}\right] \end{array} \end{array}$$

where $A^{\prime }=A-A_{ref}$ is the residual first-order coefficient of the bistatic range history.

The pre-filtering processed phase still contains a residual first-order coupling term (i.e., $A^{\prime }$). Prior to the following moving-target imaging steps, the residual first-order coupling effects must be eliminated completely. This is to be followed up by applying the first-order keystone transform [22]. The transform function is

(18) $$\begin{array}{c} \eta =\frac{f_{c}}{f+f_{c}}\eta_{m} \end{array}$$

where $\eta_{m}$ is the new azimuth time after the transformation.

Then the keystone transformed phase is given by

(19) $$\begin{array}{c} \begin{array}{c} \varphi_{2}\left(\eta_{m},f_{\tau }\right)=-\pi \frac{f_{\tau }^{2}}{K_{r}}-\frac{2\pi }{c}\left(f_{\tau }+f_{c}\right)R_{bs}\left(0\right)\;-\frac{2\pi }{c}A^{\prime }f_{c}\eta_{m}-\frac{\pi }{c}\frac{Bf_{c}^{2}}{\left(f_{\tau }+f_{c}\right)}\eta_{m}^{2}-\frac{\pi }{3c}\frac{Cf_{c}^{3}}{{\left(f_{\tau }+f_{c}\right)}^{2}}\eta_{m}^{3} \end{array} \end{array}$$

As we can see in Equation (19), the first-order polynomial of $\eta_{m}$ is $2\pi A^{\prime }f_{c}\eta_{m}/c$. Thus, the first-order coupling effect between the range frequency variable $f_{\tau }$ and the azimuth time variable $\eta_{m}$ is removed, i.e., the range walk has been corrected completely. The keystone transform is essentially a coordinate transformation and it changes the original azimuth time variable *η* to a new azimuth time variable $\eta_{m}$. However, the coupling effects continue to exist in the higher-order terms, i.e., the last two terms in Equation (19).

### 3.2. Range Curvature Correction

In order to correct the range curvature, first expanding the above phase term in Equation (19) into a Taylor series of $f_{\tau }$ and keeping up to second-order term yield

(20) $$\begin{array}{c} \begin{array}{c} \varphi_{3}\left(\eta_{m},f_{\tau }\right)=-\frac{2\pi f_{c}}{c}\left(R_{bs}\left(0\right)\;+A^{\prime }\eta_{m}+\frac{B}{2}\eta_{m}^{2}\;+\;\frac{C}{6}\eta_{m}^{3}\right)-2\pi \left(\frac{R_{bs}\left(0\right)}{c}-\frac{B\eta_{m}^{2}}{2c}-\frac{C\eta_{m}^{3}}{3c}\right)f_{\tau }\\ \;-\left(\frac{\pi }{K_{r}}+\frac{\pi }{c}\frac{B}{f_{c}}\eta_{m}^{2}+\frac{\pi }{c}\frac{C}{f_{c}}\eta_{m}^{3}\right)f_{\tau }^{2} \end{array} \end{array}$$

The new range frequency rate is

(21) $$\begin{array}{c} K^{\prime }=\frac{1}{\frac{1}{K_{r}}+\frac{B}{cf_{c}}\eta_{m}^{2}+\frac{C}{cf_{c}}\eta_{m}^{3}} \end{array}$$

Thus, the range compression should be carried out after keystone transform using the following range frequency rate

(22) $$\begin{array}{c} K_{ref}^{\prime }=\frac{1}{\frac{1}{K_{r}}+\frac{{\left.B\right|}_{v_{x},v_{y}=0}}{cf_{c}}\eta_{m}^{2}\;+\;\frac{{\left.C\right|}_{v_{x},v_{y}=0}}{cf_{c}}\eta_{m}^{3}} \end{array}$$

The range compression factor is given by

(23) $$\begin{array}{c} \varphi_{rcom}\left(f_{\tau }\right)=\pi f_{\tau }^{2}/K_{ref}^{\prime } \end{array}$$

After keystone transform and range compression, the phase term in the range frequency domain is

(24) $$\begin{array}{c} \begin{array}{c} \varphi_{4}\left(\eta_{m},f_{\tau }\right)=-\frac{2\pi f_{c}}{c}\left(R_{bs}\left(0\right)\;+A^{\prime }\eta_{m}+\frac{B}{2}\eta_{m}^{2}\;+\;\frac{C}{6}\eta_{m}^{3}\right)-2\pi \left(\frac{R_{bs}\left(0\right)}{c}-\frac{B\eta_{m}^{2}}{2c}-\frac{C\eta_{m}^{3}}{3c}\right)f_{\tau } \end{array} \end{array}$$

Then, we can construct a phase factor in the azimuth-time and range-frequency domain to compensate for the residual range curvature, which is given by

(25) $$\begin{array}{c} \phi_{\mathrm{HRCMC}}\left(\eta_{m},f_{\tau };v_{x},v_{y}\right)=\exp\left\{-j\pi \left(\frac{B\eta_{m}^{2}}{c}+\frac{C\eta_{m}^{3}}{c}\right)f_{\tau }\right\} \end{array}$$

which is a function of target motion parameters. Then, the residual range curvature correction can be given by

(26) $$S_{5}\left(\eta_{m},f_{\tau };v_{x},v_{y}\right)\;=\;S_{4}\left(\eta_{m},f_{\tau }\right)\times \phi_{\mathrm{HRCMC}}\left(\eta_{m},f_{\tau }\right)=\exp\left\{j\varphi_{4}\left(\eta_{m},f_{\tau }\right)\right\}\times \exp\left\{-j\pi \left(\frac{B\eta_{m}^{2}}{c}+\frac{B\eta_{m}^{3}}{c}\right)f_{\tau }\right\}$$

After the residual range curvature correction, the 2-D time domain echo is

(27) $$S_{6}\left(\eta_{m},\tau ;v_{x},v_{y}\right)\;=\sin c\left[B_{r}\left(\tau -\frac{R_{bs}\left(0;x,y\right)}{c}\right)\right]\times \exp\left\{-j\frac{2\pi }{\lambda }\left[R_{bs}\left(0\right)\;+A^{\prime }\eta_{m}+\frac{B}{2}\eta_{m}^{2}\;+\;\frac{C}{6}\eta_{m}^{3}\right]\right\}$$

where $B_{r}$ is the transmitted signal bandwidth.

### 3.3. Nonlinear Spatial-Variance Compensation

First, we shall evaluate the spatial-variances of Doppler parameters. Here, we model the azimuth dependence of the Doppler centroid as a first-order polynomial of azimuth position,

(28) $$\begin{array}{c} f_{dc}\left(x,y;R_{bs}\right)=f_{dc0}\left(x_{0},y_{0};R_{bs}\right)+a\eta_{m} \end{array}$$

where $f_{dc0}\left(x_{0},y_{0};R_{bs}\right)$ is the Doppler centroid of the reference point-scatterer $P_{\mathrm{MT}}\left(x_{0},y_{0};R_{bs}\right)$ and *a* is the first-order term coefficient.

Similarly, the DFR is modeled as a second-order polynomial of azimuth position as follows

(29) $$\begin{array}{c} f_{dr}\left(x,y;R_{bs}\right)=f_{dr0}\left(x_{0},y_{0};R_{bs}\right)+b\eta_{m}+c\eta_{m}^{2} \end{array}$$

where $f_{dr0}\left(x_{0},y_{0};R_{bs}\right)$ is the DFR of the reference point-scatterer $P_{\mathrm{MT}}\left(x_{0},y_{0};R_{bs}\right)$. *b* and *c* are the first- and second-order term coefficients.

After the above analysis, the range curvature corrected data in Equation (27) is transformed into the RD domain. Then, a fourth-order filtering is performed

(30) $$\begin{array}{c} H_{F}\left(f_{\eta };v_{x},v_{y}\right)\;=\exp\left\{j\pi \left(Y_{3}f_{\eta }^{3}+Y_{4}f_{\eta }^{4}\right)\right\} \end{array}$$

where $Y_{3}$ and $Y_{4}$ are the coefficients of $H_{F}\left(f_{\eta };v_{x},v_{y}\right)$.

After the fourth-order filtering, an azimuth NLCS factor is introduced in the azimuth time domain to equalize the spatial-variant Doppler centroid as well as DFR. The NLCS factor is given as follows

(31) $$\begin{array}{c} H_{ANLCS}\left(\eta_{m};v_{x},v_{y}\right)\;=\exp\left\{j\pi \left(q_{2}\eta_{m}^{2}+q_{3}\eta_{m}^{3}+q_{4}\eta_{m}^{4}\right)\right\} \end{array}$$

where $q_{2}$, $q_{3}$ and $q_{4}$ are the coefficients of the NLCS operator.

Then, the azimuth phase is transformed into the frequency domain,

(32) $$\begin{array}{c} \begin{array}{c} \phi_{azANLCS}\left(f_{\eta }\right)\;\approx D\left(f_{\eta }\right)+E\eta_{m}f_{\eta }+F\eta_{m}^{2}f_{\eta }+G\eta_{m}f_{\eta }^{2}+H\eta_{m}^{2}f_{\eta }^{2}+I\eta_{m}f_{\eta }^{3}+J\left(\eta_{m}\right) \end{array} \end{array}$$

where *E*, *F*, *G*, *H*, *I* are the expanding coefficients of the coupling term between $\eta_{m}$ and $f_{\eta }$.

In order to eliminate the azimuth variances of Doppler coefficients, the coefficient of first-order coupling between $f_{\eta }$ and $\eta_{m}$ is set to π/β, *β* is a constant scaling factor, which determines the azimuth position of the targets. Furthermore, the coefficients of other coupling terms are set to zero. Thus, we obtain an equation system with five unknowns

(33) $$\begin{array}{c} {}[\left\{\begin{array}{c} E=-\pi /\beta \\ F=0\\ G=0\\ H=0\\ I=0 \end{array}\right. \end{array}$$

Solving Equation (33), the parameters of fourth-order filtering and NLCS are given by

(34) $$\begin{array}{cc} & q_{2}=-2a\beta +\left(2\beta -1\right)f_{dr0}\\ & Y_{3}=\frac{b\left(2q_{2}+a+f_{dr0}\right)-f_{d3}\left(a+q_{2}\right)}{3{\left(f_{dr0}-a\right)}^{2}q_{2}f_{dr0}}\\ & q_{3}=\frac{2b\left(q_{2}+a\right)\left(q_{2}+f_{dr0}\right)-f_{d3}{\left(a+q_{2}\right)}^{2}-q_{2}N}{3{\left(f_{dr0}-a\right)}^{2}}\\ & Y_{4}=\frac{L/6-M(a-f_{dr0})/4}{{\left(f_{dr0}-a\right)}^{2}q_{2}f_{dr0}^{2}\left(q_{2}+f_{dr0}\right)}\\ & q_{4}=\frac{M/4-\left(f_{dr0}-a\right)f_{dr0}^{3}q_{2}Y_{4}}{a-f_{dr0}} \end{array}$$

where *L*, *M* and *N* are given by

$$\begin{array}{cc} & N=b\left(2q_{2}+a+f_{dr0}\right)-f_{d3}\left(a+q_{2}\right)\\ & \begin{array}{c} L=-\left[c{\left(q_{2}+f_{dr0}\right)}^{2}-b^{2}\left(q_{2}+f_{dr0}\right)\right]-3f_{d3}b\left(a+q_{2}\right)\\ -3q_{3}b\left(q_{2}-2f_{dr0}+3a\right)+3Y_{3}q_{2}bf_{dr0}\left(3f_{dr0}q_{2}-2aq_{2}+f_{dr0}a\right) \end{array}\\ & M=-3f_{d3}b+3Y_{3}q_{2}bf_{dr0}^{2}-3q_{3}b \end{array}$$

After the extended NLCS process, the signal phase in the frequency domain is given by

(35) $$\begin{array}{c} \begin{array}{c} \phi_{az\_last}\left(f_{\eta };v_{x},v_{y}\right)=-\frac{\pi }{\beta }\eta_{m}f_{\eta }-\frac{\pi f_{\eta }^{2}}{q_{2}+f_{dr0}}+\pi \frac{\left(f_{d3}/3+Y_{3}f_{dr0}^{3}+q_{3}\right)f_{\eta }^{3}}{{\left(q_{2}+f_{dr0}\right)}^{3}}+\pi \frac{\left(Y_{4}f_{dr0}^{4}+q_{4}\right)f_{\eta }^{4}}{{\left(q_{2}+f_{dr0}\right)}^{4}} \end{array} \end{array}$$

Compared with the existing NLCS algorithms in [23,24], here the azimuth chirp scaling processing is conducted together with a fourth-order filtering in Equation (30). This combination can increase two freedom degrees in the azimuth phase term, which is necessary for the balance of the nonlinear spatial-variances of the Doppler parameters in this paper.

### 3.4. Azimuth Compression

After the above spatial-variant doppler parameter compensation, the azimuth compression function can be given by

(36) $$H_{AC}\left(f_{\eta };v_{x},v_{y}\right)=\exp\left\{j\frac{\pi f_{\eta }^{2}}{q_{2}+f_{dr0}}\right\}\times \exp\left\{-j\pi \frac{\left(f_{d3}/3+Y_{3}f_{dr0}^{3}+q_{3}\right)f_{\eta }^{3}}{{\left(q_{2}+f_{dr0}\right)}^{3}}\right\}\times \exp\left\{-j\pi \frac{\left(Y_{4}f_{dr0}^{4}+q_{4}\right)f_{\eta }^{4}}{{\left(q_{2}+f_{dr0}\right)}^{4}}\right\}$$

which is a high-order polynomial function.

Then, the azimuth compression processing can be given by

(37) $$\begin{array}{c} S_{\mathrm{DE}}\left(\eta_{m},\tau ;v_{x},v_{y}\right)={\mathrm{IFT}}_{az}\left[S_{az\_last}\left(f_{\eta },\tau ;v_{x},v_{y}\right)\times H_{AC}\left(f_{\eta },\tau ;v_{x},v_{y}\right)\right] \end{array}$$

where ${\mathrm{IFT}}_{az}\left[\cdot \right]$ is the IFT over the azimuth frequency $f_{\eta }$, $S_{az\_last}\left(f_{\eta },\tau ;v_{x},v_{y}\right)=\exp\left\{j\phi_{az\_last}\left(f_{\eta },\tau ;v_{x},v_{y}\right)\right\}$ is the echo signal after the NLCS process.

### 3.5. Motion Parameter Estimation

Since the above three processes (i.e., residual range curvature correction in Equation (26), NLCS process in Equations (30) and (31), and azimuth compression in Equation (37)) are all related to the target motion parameters, then the processing result $S_{\mathrm{DE}}\left(\eta_{m},\tau ;v_{x},v_{y}\right)$ in Equation (37) is a function of $v_{x}$ and $v_{y}$, which should be estimated here.

#### 3.5.1. Transforming the Imaging Problem to Be a New Optimization Problem

In order to solve the above issues, the imaging problem here is transformed to be a new optimization problem, whose optimal criterion is the local minimum entropy [25,26]. Minimum entropy mainly states that any inference results made should be based on the probability distribution naturally [25]. Inference should have the minimum entropy that is permitted by data taken during observation. That is to say, the moving target is well focused when its image entropy is minimum. Firstly, the new optimization problem can be given by

(38) $$\begin{array}{c} \left\{\begin{array}{c} {\hat{v}}_{\mathrm{opt}}=\arg\min_{v}\underset{\Omega }{\;\int \int \;}\left\{-\rho \left(\eta_{m},\tau ;v\right)\log\rho \left(\eta_{m},\tau ;v\right)\right\}d\Omega \\ s.t.\;\;v_{x}\in \left(v_{x\min},v_{x\max}\right),\;v_{y}\in \left(v_{y\min},v_{y\max}\right) \end{array}\right. \end{array}$$

where

(39) $$\begin{array}{c} \rho \left(\eta_{m},\tau ;v\right)\;=\;\frac{{\left|S_{\mathrm{DE}}\left(\eta_{m},\tau ;v_{x},v_{y}\right)\right|}^{2}}{\underset{\Omega }{\;\int \int \;}{\left|S_{\mathrm{DE}}\left(\eta_{m},\tau ;v_{x},v_{y}\right)\right|}^{2}d\eta_{m}d\tau } \end{array}$$

and $v=[v_{x},v_{y}]$, $v_{x\min}$ and $v_{x\max}$ are the minimum and maximum bounds of $v_{x}$, $v_{y\min}$ and $v_{y\max}$ are the minimum and maximum bounds of $v_{y}$.

#### 3.5.2. Solving the New Optimization Problem Based on Differential Evolution

Differential Evolution (DE) is arguably one of the most powerful stochastic real-parameter optimization algorithms in current use [27]. DE proposed by Storn and Price [28] is a population based evolutionary algorithm for real parameter optimization. DE starts by randomly initializing the population to cover the entire search space uniformly. The individuals of the population are then perturbed and combined by applying mutation and crossover operators to produce new candidates. The new population is generated by selecting the better individuals among the current population and the new candidates. The overall procedure repeats until the stopping criteria is satisfied.

Let $v_{i,G}=\left(v_{x,i,G},v_{y,i,G}\right)$ represent the *i*th individual of the population at the *G*th generation. The size of the population is denoted as *N*. The solving processing of DE includes the following steps:

*Step* 1: Initialize the population based on the minimum and maximum bounds,
  *v*_*x*,*i*,0_ = *v*_*x*,min_ + rand_*ij*_ [0, 1] × (*v*_*x*,max_ − *v*_*x*,min_)
  *v*_*y*,*i*,0_ = *v*_*y*,min_ + rand_*ij*_ [0, 1] × (*v*_*y*,max_ − *v*_*y*,min_)
*Step* 2: Randomly select three mutually distinct vectors from the population $v_{r_{1}^{i}G},v_{r_{2}^{i}G},v_{r_{3}^{i}G}$;
*Step* 3: Create the donor vector for the ith individual,
  $$\Gamma_{i,G}=v_{r_{1}^{i}G}+p\times (v_{r_{2}^{i}G}-v_{r_{3}^{i}G})$$
*Step* 4: Generate the trail vector $u_{i,G}$ through the following crossover operator,
  $$u_{i,j,G}=\{\begin{array}{c} \Gamma_{j,i,G}\;\text{if}\;({\text{rand}}_{i,j}[0,1]\leq C_{r}\;\text{or}\;j=j_{rand})\\ v_{j,i,G}\;\text{otherwise} \end{array}$$
*Step* 5: Correct the residual range curvature and compensate the spatial-variant Doppler parameters of the moving target through Equations (26), (30) and (31) using $v_{i,G}$ and $u_{i,G}$, separately;
*Step* 6: Azimuth compression through Equation (37) using $v_{i,G}$ and $u_{i,G}$, separately;
*Step* 7: Compute the local image entropies around the moving target of the two images $S_{\mathrm{DE}1}$ and $S_{\mathrm{DE}2}$,
  $$H_{S_{\mathrm{DE}1,G}}=-\iint_{\Omega }\{\rho (\eta_{m},\tau ;v_{i,G})\text{log}\rho (\eta_{m},\tau ;v_{i,G})\}d\eta_{m}d\tau$$
  $$H_{S_{\mathrm{DE}2,G}}=-\iint_{\Omega }\{\rho (\eta_{m},\tau ;u_{i,G})\text{log}\rho (\eta_{m},\tau ;u_{i,G})\}d\eta_{m}d\tau$$
*Step* 8: Produce this generation through the following selector,
  $$v_{i,G+1}=\{\begin{array}{cc} u_{i,G} & \text{if}\;H_{S_{\mathrm{DE}1,G}}>H_{S_{\mathrm{DE}2,G}}\\ v_{i,G} & \text{otherwise} \end{array}$$
*Step* 9: Continue the above *Steps* until the local minimum entropy is obtained,
  $${\hat{v}}_{\mathrm{opt}}=v_{i,k}\text{ when }|H_{S_{\mathrm{DE},k}}-H_{S_{\mathrm{DE},\mathrm{minimum}}}|<e$$
  where *e* represents a very small error, such as 10^−2^.

After the new optimization problem has been solved by DE, not only can the residual range curvature be completely corrected, but also the nonlinear spatial-variances of the Doppler parameters can be compensated by the adaptive NLCS accurately. Finally, the geometric rectification for the imaging result from the bistatic range domain to the scene domain should be done via the relationship between the bistatic range $R_{bs}$ and the *x*-axis coordinate. The relationship can be given by [18]

(40) $$\begin{array}{c} x\left(R_{bs},y\right)=\frac{-N+\sqrt{N^{2}-4MP}}{2M} \end{array}$$

where

$$\begin{array}{cc} & M\;=\;A^{2}-4R_{bs}^{2}\\ & N\;=\;2ABy+2AC+8R_{bs}^{2}x_{T}\\ & P\;=\;B^{2}y^{2}+C^{2}+2BCy-4R_{bs}^{2}x_{T}^{2}-4R_{bs}^{2}y_{T}^{2}+8R_{bs}^{2}y_{T}y-4R_{bs}^{2}y^{2}-4R_{bs}^{2}z_{T}^{2}\\ & A\;=\;2x_{T}-2x_{R}\\ & B\;=\;2y_{T}-2y_{R}\\ & C\;=\;x_{R}^{2}+y_{R}^{2}+z_{R}^{2}-x_{T}^{2}-y_{T}^{2}-z_{T}^{2}-R_{bs}^{2} \end{array}$$

**Figure 2:**
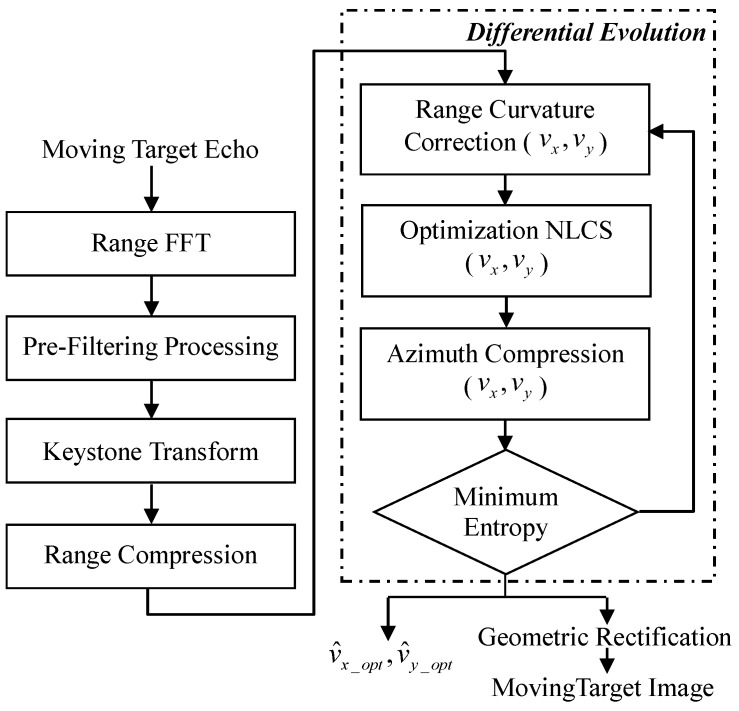
The flowchart of the proposed method.

Figure 2 shows the flowchart of the proposed BFSAR moving-target imaging method.

## 4. Computational Complexity

Suppose that the range sample number is denoted as $N_{r}$ and the azimuth sample number is $N_{a}$. The total number of real floating-point operations is

(41) $$\begin{array}{c} 10N_{r}N_{a}\log_{2}\left(N_{r}\right)+20N_{r}N_{a}\log_{2}\left(N_{a}\right)+36N_{r}N_{a}+2\left(2M_{key}-1\right)N_{r}N_{a} \end{array}$$

where $M_{key}$ is the number of neighbor samples used for the azimuth interpolation. Therefore, the computational complexity is of order $O\left(N^{2}\log_{2}N\right)$, where *N* is the 1-D size of the data.

## 5. Numerical Simulations

**Figure 3:**
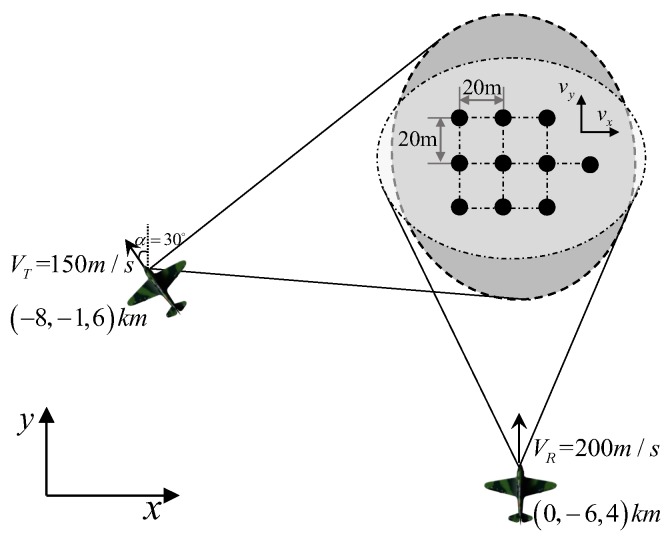
The original simulated moving-target scene.

To verify the validity of the proposed BFSAR moving-target imaging method, numerical simulations are carried out in this section. The relevant simulation parameters are shown in Table 1. To highlight the moving-target imaging capability of the proposed technology, the target is assumed to comprise ten point-scatterers, and it is shown in Figure 3. The distances between each two adjacent point-scatterers along the x-axis and the y-axis are both 20 m. The cross-track velocity of the moving target (i.e., $v_{x}$) is assumed to be 12 m/s and the along-track velocity (i.e., $v_{y}$) 10 m/s.

**Figure 4:**
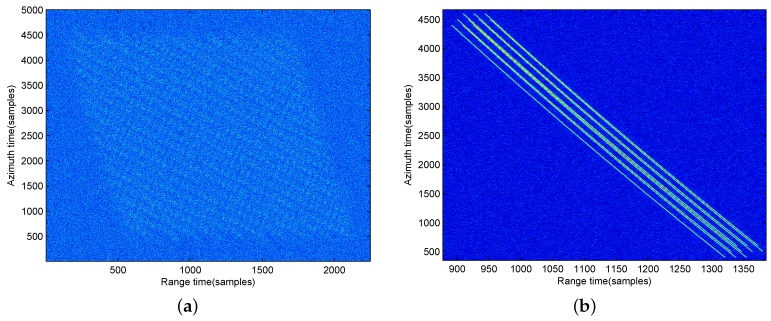
Raw date of the moving target for BFSAR. (**a**) In the 2-D time domain; (**b**) In the range-compressed domain.

**Figure 5:**
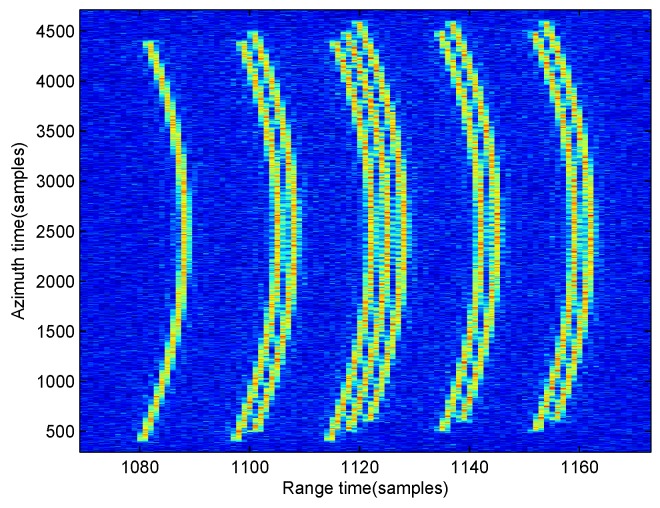
Keystone transform processed data in the range-compressed domain.

First, moving-target raw data is shown in Figure 4, which is blurry in noise with SNR =−10 dB. Figure 4a is the 2-D time domain raw data , and Figure 4b is the range-compressed domain raw data. The keystone transform processed image is shown in Figure 5. By making a comparison between Figure 4 and Figure 5, we can observe that the range walk of the moving target has been corrected by the keystone transform, but the high-order RCM still exists in Figure 5 (moving-target energy still spreads in several range gates since the effect of the high-order RCM, that should be corrected before the azimuth compression).

**Figure 6:**
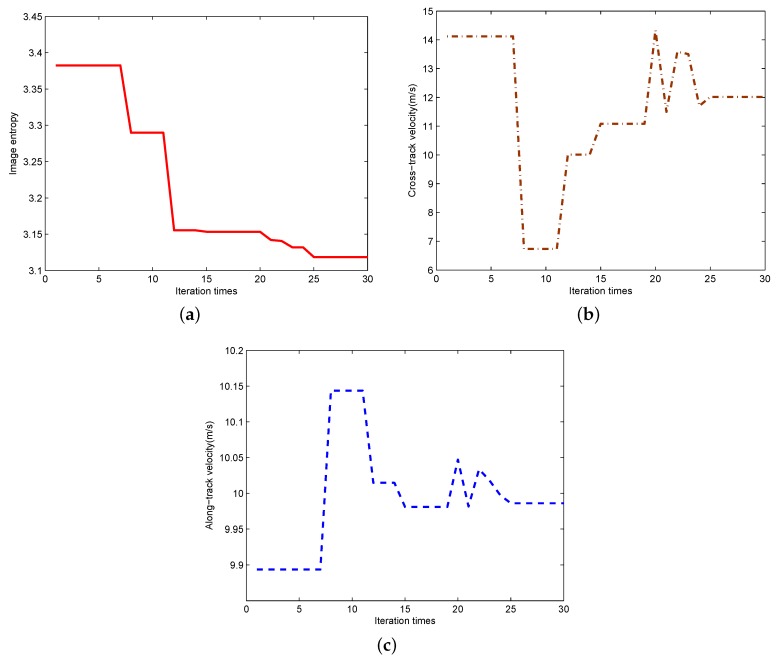
The solving process of the new optimization problem for the moving-target imaging. (**a**) Image entropy; (**b**) Estimated cross-track velocity; (**c**) Estimated along-track velocity.

During DE to solve the new optimization problem, minimum and maximum bounds of $v_{x}$ and $v_{y}$ are set to be −30 m/s and 30 m/s, respectively. In addition, population size *N* is set to be 100. The solving process of the new optimization problem based on DE is shown in Figure 6, where Figure 6a shows the image entropy changes along with different iteration times, Figure 6b,c shows the estimated cross-track velocity and along-track velocity, respectively. Based on the four subfigures, we can observe that the search-processing has high efficiency since after 25 iterations, it begins to converge. In addition, after the new optimization problem has been solved by DE, the optimal solution of v is ${\hat{v}}_{\mathrm{opt}}$ = [12.0167 m/s, 9.9861 m/s], i.e., ${\hat{v}}_{x}=12.0167$ m/s and ${\hat{v}}_{y}=9.9861$ m/s. Compared with the theoretical values v= [12 m/s, 10 m/s], we can observe that the estimation errors of the velocity parameters are less than 0.14%.

**Figure 7:**
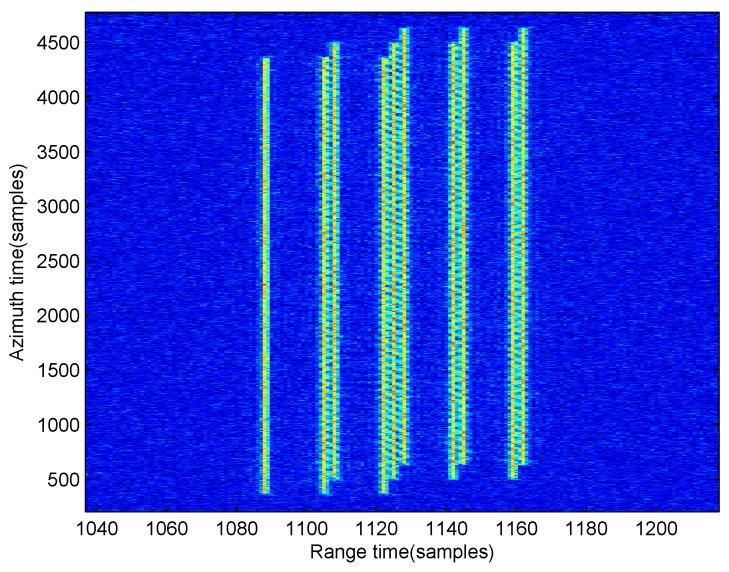
Range curvature corrected image for the moving target.

Using the optimal solution v is ${\hat{v}}_{\mathrm{opt}}=$ [12.0167 m/s, 9.9861 m/s], the range curvature of the moving target can be corrected completely using Equation (26), and the processed result is shown in Figure 7. From this figure, we can establish that most moving target energy has been gathered in one range bin.

**Figure 8:**
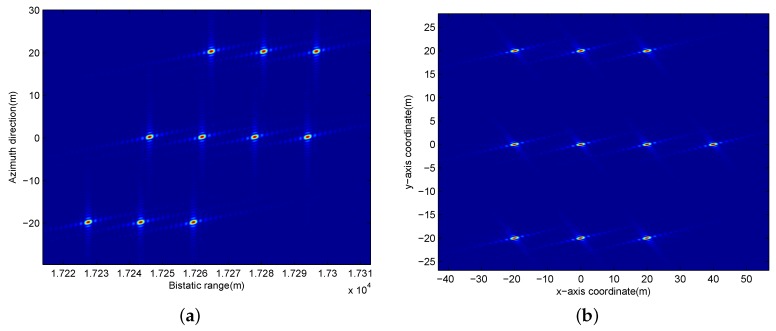
Imaging result of the moving target for BFSAR. (**a**) Before geometric rectification; (**b**) After geometric rectification.

Finally, the moving target can be well refocused and the imaging result is shown in Figure 8, where Figure 8a is before the geometric rectification and its range direction is in the bistatic range domain, while Figure 8b is after geometric rectification and it is in the same domain with the original scene in Figure 3.

## 6. Conclusions

This paper presents an adaptive moving-target imaging method for BFSAR. The validity of this method is verified by numerical simulations with detailed analysis. This method relies on a proper processing of the data aiming at, first, to correct the range walk by applying keystone transform over the whole received echo; then, the relationships between unknown high-order RCM, nonlinear spatial-variances of the Doppler parameters, and speed of the mover, are established. After that, using an optimization NLCS technique, not only can the unknown high-order RCM be accurately corrected, but also the nonlinear spatial-variances of the Doppler parameters can be balanced. Finally, a high-order polynomial filter is applied to compress the whole azimuth data of the moving target. The simulation results show that the proposed method has high estimation precision.

## Figures and Tables

**Table 1:** Relevant Parameters of the Simulations.

Parameter	Value
Center frequency	9.6 GHz
Range bandwidth	200 MHz
PRF	1000 Hz
GMT center Location	(0,0,0) m
Transmitter Location	(−8,−1,6) km
Receiver Location	(0,−6,4) km
Receiver’s Velocity	200 m/s
Transmitter’s Velocity	150 m/s
Angle *α*	$30^{\circ }$
